# Association of opioid receptor gene polymorphisms with drinking severity and impulsivity related to alcohol use disorder in a Korean population

**DOI:** 10.1111/cns.13138

**Published:** 2019-04-19

**Authors:** Chun Il Park, Syung Shick Hwang, Hae Won Kim, Jee In Kang, Sang Hak Lee, Se Joo Kim

**Affiliations:** ^1^ Institute of Behavioral Science in Medicine Yonsei University College of Medicine Seoul Republic of Korea; ^2^ Graduate School Yonsei University College of Medicine Seoul Republic of Korea; ^3^ Department of Psychiatry Yonsei University College of Medicine Seoul Republic of Korea; ^4^ Department of Medical Education Yonsei University College of Medicine Seoul Republic of Korea; ^5^ Division of Cardiology, Department of Internal Medicine, Severance Hospital Yonsei University College of Medicine Seoul Republic of Korea; ^6^ Cardiovascular Research Institute Yonsei University College of Medicine Seoul Republic of Korea

**Keywords:** alcohol use disorder, drinking severity, impulsivity, opioid receptor gene

## Abstract

**Aims:**

Recent evidence suggests that the opioid system is implicated in the pathophysiology of alcohol use disorder (AUD). We aimed to examine the genetic influence of opioid receptors on susceptibility to AUD and its clinical and psychological characteristics including harmful drinking behavior and various aspects of impulsivity in AUD patients.

**Methods:**

Three μ‐opioid receptor gene (*OPRM1*) variants and two κ‐opioid receptor gene (*OPRK1*) variants were examined in 314 male patients with AUD and 324 male controls. We applied the Alcohol Use Disorders Identification Test (AUDIT), Obsessive Compulsive Drinking Scale (OCDS), and Alcohol Dependence Scale. AUD patients also completed the stop‐signal task, delay discounting task, balloon analogue risk task, and the Barratt Impulsiveness Scale version 11 (BIS‐11).

**Results:**

No significant differences in genotype distributions or haplotype frequencies were found between AUD patients and controls. However, *OPRK1* SNP rs6473797 was significantly related to the severity of alcohol‐related symptoms as measured by AUDIT and OCDS and a haplotype containing rs6473797 was also related to OCDS scores in AUD patients. For other psychological traits, *OPRM1* SNP rs495491 was significantly associated with scores on the motor subfactor of the BIS‐11.

**Conclusion:**

Genetic variations in opioid receptors may contribute to symptom severity and impulsivity in AUD patients.

## 1 INTRODUCTION

Alcohol use disorder (AUD) is a complex illness involving multiple genetic and environmental factors. Based on studies of twins and adoption, the hereditary component of alcohol dependence has been estimated at 50%‐60%.^1^ The endogenous opioid system has been implicated in the development of alcohol dependence due to its prominent role in the central reward mechanism.^2^ Existing studies suggest that the level of alcohol‐dependent activation in endogenous opioid transmission might be in part genetically determined.^3^ Among opioid receptor genes, *OPRM1* encoding the μ‐opioid receptor is the most intensively studied in relation to drug dependence and alcoholism. Several studies on the effect of single nucleotide polymorphisms (SNPs) in *OPRM1*, such as rs1799971, on alcohol dependence report contradictory results.^4^, ^5^ Furthermore, the role of the κ‐opioid receptor in the regulation of reward stimuli via the modulation of dopaminergic tone^8^ has prompted research on the association of *OPRK1* —which encodes the κ‐opioid receptor—with alcoholism.^9^ However, the role of *OPRK1* in AUD is unclear due to conflicting results.^10^, ^11^

Apart from the influence of genetic factors on the development of AUD, researchers have also sought to identify markers associated with the severity of AUD symptoms.^12^ However, few studies have explored the genetic effects of opioid receptor genes on symptom severity in AUD. In a study investigating the association of the *OPRM1* variant rs1799971 with alcoholism severity, no significant results emerged from questionnaire scores.^13^ The μ‐opioid receptor is known to play a crucial role in modulating the reinforcement effects of substances by reward circuits^14^; it is therefore valuable to investigate the link between *OPRM1* and AUD severity. The *OPRK1* gene is known to be associated with levels of alcohol use in patients with heroin dependence undergoing methadone maintenance treatment.^15^ Given the absence of studies investigating the direct genetic influence of *OPRK1* on alcohol severity in the AUD, additional research is necessary. A number of questionnaires have been designed to measure symptom severity in AUD; the Alcohol Use Disorders Identification Test (AUDIT) is considered a useful tool to identify harmful drinking.^16^ The Alcohol Dependence Scale (ADS) is one of the most broadly used instruments to assess the severity of alcohol dependence,^17^ and the Obsessive Compulsive Drinking Scale (OCDS) has been widely used to measure the severity of cravings.^18^

Previous studies in humans have established that impulsivity is closely related to alcohol problems.^19^ Impulsivity as measured in prospective studies has been shown to predict the development of AUD^20^ and to mediate the relationship between parental substance‐use disorders and the eventual development of substance‐use disorders in offspring.^21^ There is growing consensus that impulsivity is heterogeneous, involving a variety of behaviors and processes. The Barratt Impulsiveness Scale (BIS) is a well‐validated self‐report questionnaire that measures multidimensional impulsivity consisting of motor, attentional (cognitive), and nonplanning impulsivity.^22^ Several behavioral tasks have also been developed and used for performance‐based measurement of impulsivity. In terms of behavioral disinhibition, the stop‐signal task (SST) is commonly used; in a previous study using SST, the patients with alcohol dependence showed impaired inhibitory control.^23^ The delay discounting task (DDT) is a well‐known modality to assess the tendency to discount future rewards in relation to impulsive decision making.^24^ In relatively recent years, poor outcomes resulting from risky behavior have been considered an important factor in impulsivity; the Balloon Analogue Risk Task (BART) is being used to measure the dimension of risk‐taking propensity.^25^ It is important to understand the role of impulsivity in AUD by comprehensively considering the various dimensions of impulsivity using self‐rating measures and various performance results. From the perspective of linkage in impulsivity and opioid receptor genes, the *OPRM1* variant rs1799971 has shown association with impulsivity^26^; moreover, *OPRM1* ‐knockout mice exhibited a marked reduction in motor impulsivity.^27^ In addition, the occurrence of impulsivity in patients with Parkinson's disease receiving dopamine replacement therapy is known to be related to *OPRK1* genotypes^28^; however, evidence of a relationship between opioid receptor genes and impulsivity is still lacking.

The aim of our study was to investigate the potential role of *OPRM1* and *OPRK1* in susceptibility to AUD, as well as in its symptom severity characteristics using AUDIT, OCDS, and ADS in a Korean population. In addition, we explored the association between multidimensional impulsivity and *OPRM1* and *OPRK1* using questionnaires, BIS‐11, and behavioral tasks including SST, DDT, and BART in patients with AUD. We hypothesize that opioid receptor gene polymorphisms are associated with symptom severity and impulsivity in AUD patients.

## 2 METHODS

### 2.1 Subjects

The present study included only Korean males. The patient group consisted of 314 patients with AUD who were hospitalized at the 16 psychiatric hospitals throughout Korea. They were all diagnosed as having alcohol dependence by trained psychiatrists according to the Diagnostic and Statistical Manual of Mental Disorders, 4th Edition (DSM‐IV) criteria and had been abstinent for at least one week. All subjects scored above the cutoff score of 8 on the AUDIT, which is indicative of hazardous drinking.^29^ Exclusion criteria were as follows: (a) physical or mental illness that would interfere with task performance; (b) history of major psychiatric disorder including schizophrenia and other psychotic disorders, mood disorders, or anxiety disorders; (c) history of other substance dependence in the last 6 months; and (d) a score of less than 26 on the MMSE‐K (Mini‐Mental State Examination—Korean version). For the control group, a total of 324 nonalcoholic healthy males were enrolled from the Cardiovascular Genome Center at Yonsei University College of Medicine in the Republic of Korea between November 2000 and March 2011. They visited Severance Hospital, Yonsei University Health System, for health check‐ups. They did not have any specific medical conditions. Participants provided written informed consent according to the procedures approved by the Severance Hospital Institutional Review Board, and all methods were carried out in accordance with the approved guidelines.

### 2.2 Measurements

#### 2.2.1 Questionnaires

The severity of symptoms in patients with AUD was assessed using AUDIT,^16^ OCDS,^18^ and ADS.^17^ The AUDIT consists of questions regarding alcohol consumption and the resulting consequences of drinking, with higher scores designating more harmful drinking behavior. The OCDS assesses the efforts and ability to resist thoughts of alcohol and the impulse to drink, with higher scores indicating higher craving intensities. ADS evaluates self‐administered compulsive drinking, problematic drinking behavior, and alcohol withdrawal symptoms. The severity of depressive and anxiety symptoms was assessed using the Beck Depression Inventory (BDI)^30^ and Beck Anxiety Inventory (BAI).^31^ All questionnaires were translated into Korean versions. Impulsivity was measured using the Korean version of the BIS‐11 for which we analyzed total scores as well as three subfactors including motor, cognitive, and nonplanning scores.^32^

#### 2.2.2 Behavior tasks

We conducted the SST to assess the response inhibition and stop‐signal reaction time (SSRT). A longer SSRT value indicates a slower inhibitory response. To assess the impulsivity in a choice situation, the DDT was performed by participants. We calculated the k value, with higher k values designating higher sensitivity to delayed rewards. Lastly, the BART was conducted to measure risk‐taking propensity (a value of BART). A larger adjusted value of BART represents a higher risk‐taking propensity. (See the Appendix S1 for details of the behavior tasks.).

### 2.3 SNP selection and genotyping

We selected a total of six SNPs including four *OPRM1* gene SNPs (rs1799971, rs495491, rs609148, and rs648893) and two *OPRK1* gene SNPs (rs6473797 and rs702764). In selecting the SNPs, we reviewed previous genetic studies on alcohol dependence and verified significant association with alcohol use based on SNP or haplotype analyses^4^, ^6^, ^11^, ^33^, ^34^ (Table S1). The minor allele frequency for each selected SNP was confirmed to be >0.05 in East Asian populations, based on the 1000 genomes project database (https://www.ncbi.nlm.nih.gov/variation/tools/1000genomes/; GRCh37.p13, Phase 3). Genomic DNA was extracted from blood. Genotyping was conducted using a single‐base primer extension assay (ABI PRISM^®^ SNaPshot™ Multiplex kit; ABI/Life Technologies/Thermo Fisher Scientific) according to the manufacturer's instructions.

### 2.4 Statistical analyses

Statistical analyses were performed using descriptive statistics for the demographic variables. Genotypes for rs609148 and rs648893 were perfectly linked; therefore, we dropped rs648893 from the analyses and the allele frequencies and association of traits for rs609148 were extended to rs648893. Differences in the allelic distribution of the five SNPs were examined using chi‐square tests. Associations between each SNP genotype and alcohol dependence status were examined using age‐adjusted multivariate logistic regression analysis. Linear regression models were used to evaluate associations between genotypes and various clinical measures of alcohol severity (AUDIT, OCDS, ADS) and impulsivity (BIS, SST, DDT, BART) in AUD subjects. Single‐marker analysis was conducted using the R package SNPassoc.^36^ We set the statistical significance level at *P* < 0.01 with Bonferroni correction for multiple comparisons (five SNPs). For the haplotype analysis, the pairwise linkage disequilibrium (LD) pattern of the *OPRM1* and *OPRK1* SNPs was estimated using Haploview v4.0 (http://www.broadinstitute.org/haploview/haploview)^37^ and haplotype blocks were determined from the solid spine of LD. The regression analysis of the haplotypes was conducted using the “haplo.score” function of the program “haplo.stats” (http://cran.r-project.org/src/contrib/Descriptions/haplo.stats.html).^38^ The linear regression analysis of SNPs and haplotypes was conducted with adjustments for age, total BDI score, and total BAI score. The haplotypes with frequencies <0.5% were excluded. For haplotype analyses, permutation adjustments were performed (n = 10 000) and simulated *P* < 0.05 was regarded statistically significant.

## 3 RESULTS

### 3.1 Sociodemographic and clinical characteristics

The sociodemographic and clinical characteristics as well as the behavioral task results of AUD patients and controls are presented in Table 1. All subjects were Korean males. Patients with AUD were significantly younger than the controls (*t* = 19.5, *P* < 0.001); therefore, we adjusted for age in subsequent analyses. All participants including AUD patients and controls were male.

**Table 1:** Sociodemographic and clinical characteristics and behavioral results of the study samples

Variables	AUD (n = 314)^a^	Controls (n = 324)^a^	*P*‐value
Age, y	48.5 ± 7.79	62.3 ± 10.0	<0.001
Education, y	13.5 ± 3.96		
Age of first drinking	19.4 ± 6.77		
Duration of AUD, y	17.5 ± 10.4		
AUDIT	26.7 ± 7.70		
OCDS	19.2 ± 7.29		
ADS	21.3 ± 10.5		
BIS			
Total	51.1 ± 11.0		
Cognitive	14.5 ± 3.27		
Motor	15.8 ± 4.47		
Nonplanning	20.7 ± 4.64		
BDI	18.9 ± 12.5		
BAI	13.5 ± 11.3		
Behavioral task			
SSRT (n = 286)	184 ± 125		
DDT (k value) (n = 285)	−3.41 ± 3.14		
BART (n = 279)	9.16 ± 34.4		

Note

ADS, Alcohol‐Dependent Scale; AUD, alcohol use disorder; AUDIT, Alcohol Use Disorders Identification Test; BAI, Beck Anxiety Inventory; BART, Balloon Analogue Risk Task; BDI, Beck Depression Inventory; BIS, Barratt Impulsiveness Scale; DDT, delayed discount test; OCDS, Obsessive Compulsive Drinking Scale; SSRT, stop‐signal reaction time.

a Mean ± standard deviation.

### 3.2 Association between *OPRM1/OPRK1* SNPs and AUD status

**Figure 1:**
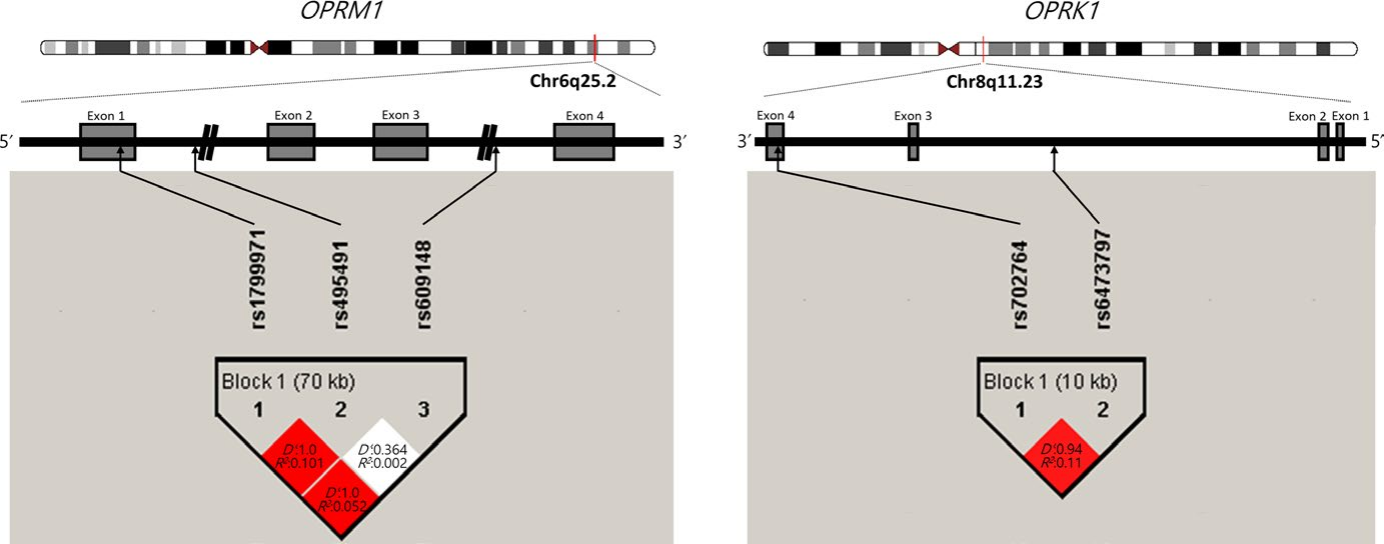
Schematic representation of the μ‐ and κ‐opioid receptor gene with the location of the SNPs analyzed in the present study, and linkage disequilibrium (LD) structure among the SNPs in healthy controls. The grey boxes indicate exons in the OPRM1 and OPRK1 genes. Haplotype blocks and LD structure were derived using Haploview version 4.2. In the haplotype blocks, pairwise D′ values and R‐squared values were presented in squares

There was no significant deviation from Hardy‐Weinberg equilibrium in the SNP data for controls, and the minor allele frequencies were >0.05 for all SNPs (Table S2). Comparison of allele and genotype distributions did not reveal significant differences between AUD cases and normal controls for all SNPs (Table 2). Two haplotype blocks were each revealed for *OPRM1* (rs1799971, rs495491, rs609148) and *OPRK1* (rs702764, rs6473797; Figure 1). The results of haplotype analysis revealed no significant differences in haplotype frequencies between patients with AUD and controls (Table S3).

**Table 2:** Distribution of allelic and genotypic frequencies of target SNPs between AUD and controls

Gene	rs number	Allele				Genotype			
		D/d^a^	AUD^b^	Control^b^	*P*‐value^c^	AUD^d^	Control^d^	OR add (95% CI)	*P*‐value^e^
*OPRM1*	rs1799971	A/G	394/230	396/250	0.499	125/144/43	127/142/54	0.86 (0.64‐1.14)	0.288
*OPRM1*	rs495491	A/G	532/92	559/89	0.607	226/80/6	245/69/10	1.20 (0.80‐1.79)	0.372
*OPRM1*	rs609148	G/A	582/40	595/49	0.413	272/38/1	273/49/0	0.89 (0.50‐1.56)	0.675
*OPRK1*	rs6473797	A/G	385/239	418/226	0.236	114/157/41	128/162/32	1.05 (0.78‐1.43)	0.746
*OPRK1*	rs702764	A/G	582/40	605/41	0.951	274/34/3	283/39/1	1.03 (0.59‐1.79)	0.931

Note

AUD, alcohol use disorder; Cl, confidence interval; OR, odds ratio; add, additive; SNP, single nucleotide polymorphism.

a Lowercase d denotes the less frequent allele.

b Number of major and minor alleles in individuals with AUD and controls.

c *P*‐values by Pearson's chi‐square test for allelic associations.

d Number of genotypes in individuals with AUD and controls. Order of genotypes: DD/Dd/dd (d is the minor allele).

e *P*‐values by multivariate logistic regression with adjustment for age.

### 3.3 Influences of *OPRM1/OPRK1* SNPs on severity of AUD

No *OPRM1* SNPs or haplotypes were related to any AUDIT, OCDS, and ADS scores. In contrast, *OPRK1* SNP rs6473797 was significantly related to the severity of alcohol‐related symptoms, as measured by the AUDIT (*P* = 0.0086) and OCDS (*P* = 0.0005; Table 3). ADS was not significantly related to rs6473797 (*P* = 0.0189) after adjusting for multiple comparisons. A haplotype containing rs6473797 (block 1, rs6473797‐rs702764) was related to the OCDS scores. The A‐A haplotype was significantly associated with lower OCDS scores (Hap‐score = −3.45, simulated [sim] *P* = 5e‐4), and the G‐A haplotype was associated with higher OCDS scores (Hap‐score = 2.60, sim *P* = 0.0088; Table 4). However, rs702764 was not significantly related to any symptom severity scores and haplotype block 1 was not significantly related to AUDIT and ADS scores (Table S4).

**Table 3:** The effects of *OPRM1* SNPs and *OPRK1* SNPs on severity of alcohol use disorder

rs number	D/d^a^	DD/Dd/dd^b^	AUDIT					OCDS					ADS				
			DD^c^	Dd^c^	dd^c^	Mean difference (95% CI)	*P* d	DD^c^	Dd^c^	dd^c^	Mean difference (95% CI)	*P* d	DD^c^	Dd^c^	dd^c^	Mean difference (95% CI)	*P* d
rs1799971	A/G	125/144/43	26.7 ± 0.71	26.4 ± 0.64	28.0 ± 1.04	0.603 (−0.54, 1.75)	0.304	19.2 ± 0.69	18.9 ± 0.59	20.4 ± 0.99	0.641 (−0.37, 1.65)	0.215	21.3 ± 0.98	20.3 ± 0.85	24.1 ± 1.45	1.01 (−0.49, 2.51)	0.188
rs495491	A/G	226/80/6	26.7 ± 0.51	26.7 ± 0.90	27.2 ± 2.28	−0.756 (−2.36, 0.84)	0.355	19.0 ± 0.46	19.6 ± 0.94	22.2 ± 2.17	−0.236 (−1.65, 1.18)	0.744	21.4 ± 0.69	20.9 ± 1.21	22.3 ± 4.81	−1.44 (−3.53, 0.65)	0.177
rs609148	G/A	272/38/1	26.7 ± 0.46	26.6 ± 1.34	36.0 ± 0.00	−0.695 (−3.00, 1.61)	0.555	19.2 ± 0.43	19.2 ± 1.37	31.0 ± 0.00	−0.691 (−2.72, 1.34)	0.505	21.3 ± 0.63	21.0 ± 1.81	41.0 ± 0.00	−0.970 (−3.98, 2.04)	0.528
rs6473797	A/G	114/157/41	25.2 ± 0.70	27.4 ± 0.60	28.6 ± 1.31	1.58 (0.41, 2.75)	**0.0086**	17.3 ± 0.64	20.1 ± 0.58	21.3 ± 1.18	1.85 (0.83, 2.87)	**0.0005**	19.6 ± 0.94	21.7 ± 0.83	24.4 ± 1.77	1.85 (0.31, 3.38)	0.019
rs702764	A/G	274/34/3	26.5 ± 0.46	28.7 ± 1.28	26.3 ± 8.84	1.08 (−1.09, 3.24)	0.331	18.9 ± 0.44	22.0 ± 1.23	20.3 ± 6.69	1.84 (−0.07, 3.74)	0.06	21.0 ± 0.63	23.6 ± 1.88	22.7 ± 7.88	1.33 (−1.51, 4.18)	0.358

Note

ADS, Alcohol‐Dependent Scale; AUDIT, Alcohol Use Disorders Identification Test; CI, confidence interval; OCDS, Obsessive Compulsive Drinking Scale; SNP, single nucleotide polymorphism. Bold values indicate statistical significance at the *P* < 0.01 level.

a Lowercase d denotes the less frequent allele.

b Number of genotypes.

c Mean ± standard error.

d *P*‐values by multivariate linear regression with adjustment for age, Beck Depression Inventory score and Beck Anxiety Inventory score.

**Table 4:** The effects of haplotype on OCDS score

Block			Hap‐Freq^a^	Hap‐Score^b^	Crude *P* c	Sim *P* d
*OPRK1* (rs6473797‐rs702764)^e^						
A	A		0.615	−3.45	0.0006	**5e‐04**
G	G		0.062	1.97	0.0493	0.0505
G	A		0.321	2.60	0.0093	**0.0088**
*OPRM1* (rs495491‐rs1799971‐rs609148)^f^						
A	A	G	0.44	−0.779	0.436	0.438
G	A	A	0.0164	−0.665	0.506	0.508
A	A	A	0.0437	−0.507	0.612	0.612
G	A	G	0.131	−0.137	0.891	0.890
A	G	G	0.364	1.26	0.209	0.21

Note

OCDS, Obsessive Compulsive Drinking Scale. Bold values indicate statistical significance at the simulated *P* < 0.05 level.

a Hap‐Freq, estimated frequency of the haplotype in the pool of all participants.

b Hap‐Score, score for the haplotype.

c Asymptotic chi‐square *P*‐value.

d Simulated *P*‐value.

e Global‐stat = 12.5, *df* = 3, *P* = 0.0057, global simulated *P* = 0.0055.

f Global‐stat = 1.87, *df* = 5, *P* = 0.867, global simulated *P* = 0.851.

### 3.4 Association between *OPRM1/OPRK1* SNPs and impulsivity in AUD patients

Only rs495491 showed significant association with the motor subfactor score of BIS (Table 5), while other SNPs were not significantly related to either total scores or subfactor scores of BIS. The results of the SNP analysis showed no significant association between *OPRM1*/*OPRK1* SNPs and SSRT calculated from the SST and k values from the DDT and BART representing multidimensional impulsivity (Table 6). No haplotypes were related to BIS total and subfactor scores or behavioral task results (Table S5).

**Table 5:** The effects of *OPRM1* SNPs and *OPRK1* SNPs on motor subfactor score of BIS

rs number	D/d^a^	DD/Dd/dd^b^	DD^c^	Dd^c^	dd^c^	Mean difference(95% CI)	*P* d
rs1799971	A/G	125/144/43	15.9 ± 0.37	15.6 ± 0.39	16.3 ± 0.74	0.17 (−0.48, 0.82)	0.611
rs495491	A/G	226/80/6	16.1 ± 0.31	15 ± 0.44	17.3 ± 1.26	−1.20 (−2.10,−0.30)	**0.0095**
rs609148	G/A	272/38/1	15.8 ± 0.27	16.2 ± 0.82	19.0 ± 0.00	−0.096 (−1.40, 1.22)	0.886
rs6473797	A/G	114/157/41	15.8 ± 0.44	15.7 ± 0.34	16.3 ± 0.70	−0.055 (−0.73, 0.62)	0.873
rs702764	A/G	274/34/3	15.7 ± 0.27	16.5 ± 0.83	17.7 ± 3.53	0.485 (−0.75, 1.72)	0.44

Note

BIS, Barratt Impulsiveness Scale; CI, confidence interval; SNP, single nucleotide polymorphism. The bold value indicates statistical significance at the *P* < 0.01 level.

a Lowercase d denotes the less frequent allele.

b Number of genotypes.

c mean ± standard error.

d *P*‐values by multivariate linear regression with adjustment for age, Beck Depression Inventory score and Beck Anxiety Inventory score.

**Table 6:** The effects of *OPRM1* SNPs and *OPRK1* SNPs on impulsivity behavioral task results

rs number	D/d^a^	SSRT						DDT (k value)						BART					
		DD/Dd/dd^b^	DD^c^	Dd^c^	dd^c^	Mean difference (95% CI)	*P* d	DD/Dd/dd^b^	DD^c^	Dd^c^	dd^c^	Mean difference (95% CI)	*P* d	DD/Dd/dd^b^	DD^c^	Dd^c^	dd^c^	Mean difference (95% CI)	*P* d
rs1799971	A/G	111/127/43	168 ± 14.2	196 ± 6.87	178 ± 20.9	11.8 (−7.95, 31.6)	0.242	112/128/43	−3.33 ± 0.32	−3.45 ± 0.29	−3.56 ± 0.31	−0.08 (−0.60, 0.44)	0.761	112/125/40	30.2 ± 1.62	27.3 ± 1.45	33.2 ± 3.04	0.351 (−2.57, 3.27)	0.814
rs495491	A/G	204/73/4	182 ± 7.19	185 ± 18.9	153 ± 27.5	−4.78 (−33.7, 24.1)	0.746	206/73/4	−3.51 ± 0.21	−3.24 ± 0.42	−1.88 ± 0.93	0.247 (−0.51, 1.00)	0.52	202/71/4	29.9 ± 1.20	28.0 ± 2.08	21.2 ± 6.24	−2.32 (−6.56, 1.91)	0.283
rs609148	G/A	245/34/1	180 ± 7.87	192 ± 17.1	213 ± 0.00	7.09 (−33.5, 47.7)	0.732	248/33/1	−3.53 ± 0.19	−2.50 ± 0.69	−4.05 ± 0.00	0.669 (−0.40, 1.74)	0.220	244/31/1	29.1 ± 1.10	30.8 ± 3.09	21.9 ± 0.00	1.44 (−4.65, 7.52)	0.644
rs6473797	A/G	104/141/36	195 ± 11.5	163 ± 9.95	218 ± 20.9	−2.00 (−23.0, 19.0)	0.852	106/142/35	−3.39 ± 0.33	−3.38 ± 0.25	−3.66 ± 0.50	−0.16 (−0.71, 0.39)	0.567	105/138/34	27.6 ± 1.60	30.7 ± 1.53	28.7 ± 2.78	1.37 (−1.71, 4.44)	0.384
rs702764	A/G	248/30/2	182 ± 8.04	189 ± 9.25	165 ± 1.67	0.99 (−39.0, 41.0)	0.961	251/30/1	−3.50 ± 0.20	−2.81 ± 0.56	−2.57 ± 0.00	0.509 (−0.59, 1.61)	0.367	245/29/2	29.7 ± 1.10	26.3 ± 3.14	23.7 ± 6.21	−3.13 (−8.98, 2.73)	0.296

Note

BART, Balloon Analogue Risk Task; CI, confidence interval; DDT, delayed discount test; SNP, single nucleotide polymorphism; SSRT, stop‐signal reaction time.

a Lowercase d denotes the less frequent allele.

b Number of genotypes.

c mean ± standard error.

d *P*‐values by multivariate linear regression with adjustment for age, Beck Depression Inventory score and Beck Anxiety Inventory score.

## 4 DISCUSSION

This study investigated the associations between five SNPs of the opioid receptor genes *OPRM1* and *OPRK1* and the affected status of several aspects of alcohol symptom severity and impulsivity related to AUD in Korean male subjects.

There was no significant difference between single‐marker or haplotype distributions of *OPRM1* or *OPRK1* and AUD status. The rs1799971 variant of *OPRM1* has been the most frequently studied SNP for its association with alcohol dependence, though results have been inconsistent. Certain studies reported a significant association between rs1799971 and alcohol dependence,^4^, ^6^, ^33^ while others reported no genetic effect for this SNP.^5^, ^7^, ^39^ The most recent meta‐analysis reviewing 17 case‐control studies including nine Caucasian studies (8026 patients in total) and eight Asian studies (1587 patients in total) concluded that rs1799971 exhibited no association with alcohol dependence in either ethnicity.^40^ For the other *OPRM1* SNPs we investigated, rs495491 and rs609148, a study among 382 European American patients with alcohol dependency showed the genetic risk effect of the rs495491‐C minor allele and the protective effect of the rs609148‐T minor allele toward alcohol dependence.^35^ However, a subsequent family‐based study with 1923 European American samples did not find any association with *OPRM1* SNPs including rs495491 and rs609148.^41^ The results of our study demonstrating a lack of association correspond with these previous studies. Although few studies exist on the association between *OPRK1* and AUD, those that have been conducted report inconsistent findings. A multicenter study with a family‐based design suggested a significant association of *OPRK1* SNP rs6473797 with alcohol dependence.^11^ However, other studies showed negative results for the genetic effect of *OPRK1* in alcoholism, contrary to ours.^10^, ^34^, ^39^

Although we were unable to find any association between *OPRM1* or *OPRK1* and the development of AUD, there were several significant associations of *OPRK1* genetic variants with AUDIT and OCDS scores. The minor G allele of rs6473797 was associated with higher AUDIT scores, signifying that the AUD patients with the G allele showed higher hazardous drinking tendencies. In addition, the G allele of rs6473797 was also related to higher OCDS scores, suggesting more severe craving symptoms. Furthermore, in haplotype block 1 of the *OPRK1* gene, the G‐A haplotype of rs6473797‐rs702764 showed higher OCDS scores, while the A‐A haplotype presented significantly lower scores. This result suggests that the rs6473797 SNP and haplotype rs6473797‐rs702764 of *OPRK1* play a role in regulating the severity of craving related to obsessive‐compulsive drinking in AUD. Although it did not reach statistical significance after correction for multiple comparisons, rs6473797 showed nominally significant association (*P* = 0.0189) with ADS. The G allele was linked to increasing ADS scores, indicating heavier drinking and severe withdrawal symptoms.^42^ This tendency for the G allele of rs6473797 to be associated with higher symptom severity in ADS is consistent with the results of the AUDIT and OCDS. In summary, our results suggest that the AUD patients carrying the G allele of *OPRK1* SNP rs6473797 show more harmful drinking behavior, cravings, and withdrawal symptoms, corresponding to the experience of more painful symptoms in AUD patients. To date, there have been few studies on the *OPRK1* gene and alcohol severity. However, several animal and human studies indirectly suggest the association between the *OPRK1* gene and alcohol severity. In animal studies, *OPRK1* ‐knockout mice showed decreased oral alcohol consumption.^43^ In an alcohol‐dependent rat model, increased κ‐opioid receptor signaling caused excessive alcohol consumption during withdrawal.^44^ In humans, the T‐T‐C‐T haplotype (rs7832417‐rs16918853‐rs702764‐rs7817710) of the *OPRK1* gene showed a relationship with elevated levels of alcohol use in heroin‐dependent patients.^15^ Regarding *OPRM1*, there were no associations between any of the SNPs or haplotypes and AUDIT, OCDS, and ADS scores in our study. Previous studies on the influences of *OPRM1* SNPs on AUDIT or ADS also reported no associations.^13^, ^45^ Considering these results in conjunction with ours, it seems that *OPRM1* is not related to the severity in drinking levels or frequency that lead to harmful consequences in AUD.

With respect to impulsivity, we initially hypothesized that μ‐ and κ‐opioid receptor genes influence multidimensional impulsivity as measured by SST, DDT, BART, and self‐report questionnaire BIS‐11 in AUD patients. In the μ‐opioid receptor gene, we found that the G allele of rs495491 in the *OPRM1* gene was significantly associated with higher motor impulsivity subfactor scores of BIS‐11. A previous animal study suggested that *OPRM1* ‐knockout mice showed significantly decreased motor impulsivity as measured by premature responses on the signaled nose poke task.^27^ In a study involving PET imaging of μ‐opioid receptors, impulsivity as measured by the NEO PI‐R (NEO Personality Inventory, Revised) showed a significant positive relationship with μ‐receptor binding potential in the right anterior cingulate and adjacent medial frontal cortex, right ventral basal ganglia, and basolateral area of the right amygdala, all of which are areas involved in the pathophysiology of substance abuse.^46^ However, the instrumental measures of impulsivity such as SST, DDT, and BART were not influenced by *OPRM1* or *OPRK1* variants in this study.^46^ Courtney et al conducted a study utilizing functional magnetic resonance imaging while performing SST and explored the functional polymorphism of *OPRM1* SNP rs1799971 in alcohol dependence.^47^ The author demonstrated the difference in functional connectivity within fronto‐striatal networks according to the genotype of *OPRM1* SNP rs1799971; nonetheless, there was no significant genotypic effect on behavioral performance results for the SST, as indicated by our results. Regarding DDT and BART, most genetic studies focused on dopamine genes^48^, ^49^; there have been no reports on the relationship between *OPRM1* or *OPRK1* and DDT or BART. A study of the association between the *DRD2 Taql A* polymorphism and BIS in alcohol‐dependent patients showed a significant dopamine gene effect on impulsiveness; another study showed that *DRD2 Taql A* and *DRD4 VNTR* polymorphisms were linked to DDT results.^48^ Despite the fact that we were unable to determine the association between opioid receptor gene variants and SST, DDT, and BART, the dopamine and opioid systems interact with the reward system and impulsivity in the brain 50; therefore, it is likely that the opioid‐related genes *OPRM1* or *OPRK1* are involved in impulsivity. Further studies are necessary to determine their influence on various measures of impulsivity.

There are several limitations in the present study. First, the sample size was relatively small, potentially obscuring significant genetic associations. Second, we were unable to select all tagging SNPs covering the entire *OPRM1* and *OPRK1* genes. Studies with larger sample sizes and greater numbers of SNPs will be needed to clearly confirm the function and impact of opioid receptor genes in AUD. Third, all participants in the present study were exclusively male. To understand the effects of genetic differences in alcohol use and impulsivity comprehensively, it would be necessary to study both male and female subjects. Lastly, impulsivity using the self‐rating scale, BIS‐11, and behavior tasks involving SST, DDT, and BART was assessed only in AUD patients, not in healthy controls. We were therefore unable to determine the regression model containing the control group to define the genetic influence of *OPRM1*/*OPRK1* on impulsivity.

In conclusion, we found no associations between selected SNPs or haplotypes of *OPRM1* or *OPRK1* genes and the development of AUD in Korean males. However, certain SNPs and haplotypes of *OPRK1* were associated with hazardous drinking tendency (AUDIT scores) and craving symptoms (OCDS scores). The *OPRM1* SNP rs495491 was related to motor impulsivity as measured by the BIS‐11 in patients with AUD. These findings suggest that the opioid receptor genes may have a modulating influence on several aspects of symptom severity related to AUD and impulsivity in Korean males with AUD.

## CONFLICT OF INTEREST

The authors declare no conflict of interest.
