# Supplementary material for: Association of opioid receptor gene polymorphisms with drinking severity and impulsivity related to alcohol use disorder in a Korean population
Source: CNS Neurosci Ther. 2019 Apr 19;26(1):30–8. doi: 10.1111/cns.13138 (PMC6930822; doi:10.1111/cns.13138)
Supplement: Supplementary file 1 [file CNS-26-30-s001.docx]

**Behavior Tasks**

A. Stop Signal Task (SST) ^1^

The SST consisted of 120 total trials; in each trial, the go stimulus (the letter ‘X’ or ‘O’) was presented to participants for 1000 ms. The participants were instructed to respond as quickly and as accurately as possible to the go stimulus (‘Z’ key for ‘X’, ‘/’ key for ‘O’). The stop trials (25% of trials) presented a beeping sound as a stop signal before the go stimulus, then participants would withhold their response. The onset of the stop signal was made variable by a tracking algorithm; the stop-signal delay was initially 250 ms then decreased by 50 ms after a previous stop task failure and increased by 50 ms after a previous success. We used the following outlier criteria: (1) percent inhibition on stop trials less than 25% or greater than 75%; (2) percent go-response less than 60%; (3) percent go-errors greater than 10%; and (4) a negative or less than 50 ms stop-signal reaction time (SSRT) ^2^. The main dependent variable, SSRT, was extracted by the quantile method which does not require an assumption of 50% inhibition ^1^. In this study, we used the Korean version of the SST.

B. Delay Discounting Task (DDT) ^3^

DDT was assessed using a binary choice procedure. In each trial, participants were asked to choose either an immediate or a delayed reward of virtual money. An immediate reward was smaller, and a delayed reward was larger. The delayed reward was fixed at a Korean currency equivalent to 100 US dollars. First, the duration of delay was fixed at one week, with 26 total immediate rewards presented in decreasing order, one by one. Next for each temporal delay, the number of immediate rewards was presented in increasing order. Subsequent sessions were repeated with incrementally longer delay times of one week, two weeks, one month, six months, one year, three years, and ten years. The indifference points determined between the immediate and delayed responses at each time point were recalculated to obtain the k values ^4^.

C. Balloon Analogue Risk Task (BART) ^5^

The participants were instructed that clicking a button would inflate the balloon. At the same time, each click earned a certain monetary reward, but at some point, the balloon would pop and all earned money would be lost in each trial. A popping balloon was presented with auditory and visual effects on the monitor. Also, participants were instructed that by clicking a button marked “Collect” at any time before the balloon popped, they could earn the money permanently. The entire trial consisted of 30 balloons. Participants were instructed to imagine that the monetary reward was real and asked to try hard to earn as much money as possible. Risk-taking propensity (a value of BART) was measured by the average number of inflations over the trials in which the balloons did not explode. We used the original version of BART translated into Korean.

References

1. Band GP, van der Molen MW, Logan GD. Horse-race model simulations of the stop-signal procedure. *Acta psychologica.* 2003;112(2):105-142.

2. Congdon E, Mumford JA, Cohen JR, Galvan A, Canli T, Poldrack RA. Measurement and reliability of response inhibition. *Frontiers in psychology.* 2012;3:37.

3. Hurst RM, Kepley HO, McCalla MK, Livermore MK. Internal consistency and discriminant validity of a delay-discounting task with an adult self-reported ADHD sample. *Journal of attention disorders.* 2011;15(5):412-422.

4. Mazur JE. An adjusting procedure for studying delayed reinforcement. *Commons, ML; Mazur, JE; Nevin, JA.* 1987:55-73.

5. Lejuez CW, Read JP, Kahler CW, et al. Evaluation of a behavioral measure of risk taking: the Balloon Analogue Risk Task (BART). *J Exp Psychol Appl.* 2002;8(2):75-84.

| **Supplementary Tables**  **Table** **S1** Selection of OPRM1 and OPRK1 single-nucleotide polymorphisms (SNPs). Circles denote SNPs significantly | | | | | | | |
| --- | --- | --- | --- | --- | --- | --- | --- |
| associated with alcohol dependence in single-marker analyses or haplotype analyses of previous studies. | | | | | | | |
|  | OPRM1 | | | | OPRK1 | |  |
|  | rs1799971 | rs495491 | rs609148 | rs648893 | rs6473797 | rs702764 |  |
| Zhang H. et al (2006)^1^ |  | ○ | ○ | ○ |  |  |  |
| Xuei X. et al (2006)^2^ |  |  |  |  | ○ |  |  |
| Zhang H. et al (2008)^3^ |  |  |  |  |  | ○* |  |
| Deb I. et al (2010)^4^ | ○ |  |  |  |  |  |  |
| Koller. et al (2012)^5^ | ○ |  |  |  |  |  |  |
| Miranda R. et al (2010)^6^ | ○ |  |  |  |  |  |  |
| *Significance in haplotype analysis | | | | | | | |
| ^1^Zhang H, Luo X, Kranzler HR, Lappalainen J, Yang BZ, Krupitsky E, et al. Association between two mu-opioid receptor gene (OPRM1) haplotype | | | | | | | |
| blocks and drug or alcohol dependence. Hum Mol Genet. 2006;15: 807-819. | | | | | | | |
| ^2^Xuei X, Dick D, Flury-Wetherill L, Tian HJ, Agrawal A, Bierut L, et al. Association of the kappa-opioid system with alcohol dependence. | | | | | | | |
| Mol Psychiatry. 2006;11: 1016-1024. | | | | | | | |
| ^3^Zhang H, Kranzler HR, Yang BZ, Luo X, Gelernter J. The OPRD1 and OPRK1 loci in alcohol or drug dependence: OPRD1 variation modulates | | | | | | | |
| substance dependence risk. Mol Psychiatry. 2008;13: 531-543. | | | | | | | |
| ^4^Deb I, Chakraborty J, Gangopadhyay PK, Choudhury SR, Das S. Single‐nucleotide polymorphism (A118G) in exon 1 of OPRM1 gene causes | | | | | | | |
| alteration in downstream signaling by mu‐opioid receptor and may contribute to the genetic risk for addiction. Journal of neurochemistry.  2010;112: 486-496. | | | | | | | |
| ^5^Koller G, Zill P, Rujescu D, Ridinger M, Pogarell O, Fehr C, et al. Possible association between OPRM1 genetic variance at the 118 locus and | | | | | | | |
| alcohol dependence in a large treatment sample: relationship to alcohol dependence symptoms. Alcohol Clin Exp Res. 2012;36: 1230-1236. | | | | | | | |
| ^6^Miranda R, Ray L, Justus A, Meyerson LA, Knopik VS, McGeary J, et al. Initial evidence of an association between OPRM1 and adolescent alcohol | | | | | | | |
| misuse. Alcoholism: clinical and experimental research. 2010;34: 112-122. | | | | | | | |

| **Table** **S2** Characteristics of the *OPRK1* and *OPRM1* variants. | | | | | | | | | | | |  |  | |
| --- | --- | --- | --- | --- | --- | --- | --- | --- | --- | --- | --- | --- | --- | --- |
| Gene | rs number | Chr | | Position^a^ | | Geno^b^ | | *P*_HWE_^c^ | | MAF | Function | | |  |
| *OPRM1* | rs1799971 | 6 | | 154360797 | | 99.8 | | 0.197/0.904/0.312 | | 0.378 | Missense variant | | |  |
| *OPRM1* | rs495491 | 6 | | 154382542 | | 100 | | 0.0951/1.00/0.327 | | 0.142 | Intron variant | | |  |
| *OPRM1* | rs609148 | 6 | | 154431015 | | 99.5 | | 0.236/1.00/0.356 | | 0.071 | Intron variant | | |  |
| *OPRK1* | rs6473797 | 8 | | 54152982 | | 99.7 | | 0.0676/0.234/0.033 | | 0.367 | Intron variant | | |  |
| *OPRK1* | rs702764 | 8 | | 54142157 | | 99.7 | | 1.00/0.119/0.312 | | 0.064 | Synonymous | | |  |
| Chr, chromosome; HWE, Hardy-Weinberg equilibrium; MAF, minor allele frequency; ^a^Information on the chromosomal | | | | | | | | | | | | | | |
| position is based on NCBI genome build GRCh37.p13. The locations are in reference to NM_000912.3 for *OPRK1* | | | | | | | | | | | | | | |
| and NM_000914.3 for *OPRM1*. ^b^Genotyping call rate (%). ^c^*p*-value for Hardy-Weinberg equilibrium among controls, | | | | | | | | | | | | | | |
| individuals with alcohol use disorder, and all samples. Order of *p*-values: control subjects/alcohol use disorder | | | | | | | | | | | | | | |
| subjects/total subjects. | | |  | |  | |  | |  | | |  |  | |

| **Table** **S3** The effects of haplotype on affected status of AUD | | | | | | | | | | | |  | | |  | |
| --- | --- | --- | --- | --- | --- | --- | --- | --- | --- | --- | --- | --- | --- | --- | --- | --- |
| Block | | | | Hap-Freq^a^ | | Hap-Score^b^ | | | Crude *p*^c^ | Sim *p*^d^ | | |  |  |  |  |
| 1 (rs6473797-rs702764)^e^ | | | |  | | |  | | | |  | | |  | |  |
| A | A |  | | 0.631 | | -0.238 | | | 0.812 | 0.814 | | |  |  |  |  |
| G | G |  | | 0.0618 | | 0.20 | | | 0.841 | 0.843 | | |  |  |  |  |
| G | A |  | | 0.305 | | 0.215 | | | 0.83 | 0.834 | | |  |  |  |  |
| 2 (rs495491-rs1799971-rs609148)^f^ | | | |  | | |  | | | |  | | |  | |  |
| A | A | A | | 0.057 | | -1.23 | | | 0.22 | 0.227 | | |  |  |  |  |
| A | G | G | | 0.376 | | -1.11 | | | 0.265 | 0.272 | | |  |  |  |  |
| G | A | G | | 0.131 | | 0.568 | | | 0.57 | 0.579 | | |  |  |  |  |
| A | A | G | | 0.423 | | 0.924 | | | 0.356 | 0.363 | | |  |  |  |  |
| G | A | A | | 0.0115 | | 1.23 | | | 0.22 | 0.23 | | |  |  |  |  |
| ^a^Hap-Freq, estimated frequency of the haplotype in the pool of all participants. | | | | | | | | | | | | | | |  | |
| ^b^Hap-Score, score for the haplotype. | | | | |  | | |  | | | |  | | |  | |
| ^c^Asymptotic chi-square *p*-value. | | | | |  | | |  | | | |  | | |  | |
| ^d^Simulated *p*-value. | | |  | |  | | |  | | | |  | | |  | |
| ^e^Global-stat = 0.843, df = 3, *p* = 0.839, global simulated *p* = 0.849 | | | | | | | | | | | |  | | |  | |
| ^f^Global-stat = 5.23. df = 5, *p* = 0.388, global simulated *p* = 0.388 | | | | | | | | | | | |  | | |  | |

| **Table** **S4** The effects of haplotype on AUDIT and ADS. | | | | | | | | |  | | | |  | | | | | |  | |  | |  | |  |  | |  |
| --- | --- | --- | --- | --- | --- | --- | --- | --- | --- | --- | --- | --- | --- | --- | --- | --- | --- | --- | --- | --- | --- | --- | --- | --- | --- | --- | --- | --- |
|  | Block | | | | Hap-Freq^a^ | | Hap-Score^b^ | | | Crude *p*^c^ | | | | Sim *p*^d^ | | | | Global-stat | | df | | *p* | | Global simulated *p* | | |  |  |
| AUDIT | 1 (rs6473797-rs702764) | | | |  | |  | | | |  | | | | |  | | 7.12 | | 3 | | 0.0682 | | 0.0632 | | |  |  |
|  | A | A |  | | 0.615 | | -2.57 | | | 0.0103 | | | | 0.0104 | | | |  | |  | |  | |  | | |  |  |
|  | G | G |  | | 0.062 | | 1.10 | | | 0.273 | | | | 0.274 | | | |  | |  | |  | |  | | |  |  |
|  | G | A |  | | 0.321 | | 2.19 | | | 0.0288 | | | | 0.029 | | | |  | |  | |  | |  | | |  |  |
|  | 2 (rs495491-rs1799971-rs609148) | | | |  | |  | | |  | | | | |  | | | 1.81 | | 5 | | 0.875 | | 0.87 | | |  |  |
|  | G | A | G | | 0.131 | | -0.751 | | | 0.453 | | | | 0.455 | | | |  | |  | |  | |  | | |  |  |
|  | G | A | A | | 0.0164 | | -0.705 | | | 0.481 | | | | 0.483 | | | |  | |  | |  | |  | | |  |  |
|  | A | A | A | | 0.0437 | | -0.423 | | | 0.673 | | | | 0.672 | | | |  | |  | |  | |  | | |  |  |
|  | A | A | G | | 0.44 | | -0.192 | | | 0.848 | | | | 0.847 | | | |  | |  | |  | |  | | |  |  |
|  | A | G | G | | 0.364 | | 1.01 | | | 0.313 | | | | 0.313 | | | |  | |  | |  | |  | | |  |  |
| ADS | 1 (rs6473797-rs702764) | | | |  | | |  | | | |  | | | | |  | 5.73 | | 3 | | 0.125 | | 0.127 | | |  |  |
|  | A | A |  | | 0.615 | | -2.30 | | | 0.0216 | | | | 0.0218 | | | |  | |  | |  | |  | | |  |  |
|  | G | G |  | | 0.0622 | | 1.03 | | | 0.302 | | | | 0.304 | | | |  | |  | |  | |  | | |  |  |
|  | G | A |  | | 0.321 | | 1.92 | | | 0.0545 | | | | 0.0541 | | | |  | |  | |  | |  | | |  |  |
|  | 2 (rs495491-rs1799971-rs609148) | | | |  | | |  | | | |  | | | | |  | 3.08 | | 5 | | 0.687 | | 0.685 | | |  |  |
|  | G | A | G | | 0.131 | | -1.17 | | | 0.241 | | | | 0.244 | | | |  | |  | |  | |  | | |  |  |
|  | G | A | A | | 0.0164 | | -0.744 | | | 0.457 | | | | 0.458 | | | |  | |  | |  | |  | | |  |  |
|  | A | A | A | | 0.0437 | | -0.446 | | | 0.655 | | | | 0.656 | | | |  | |  | |  | |  | | |  |  |
|  | A | A | G | | 0.44 | | -0.161 | | | 0.872 | | | | 0.871 | | | |  | |  | |  | |  | | |  |  |
|  | A | G | G | | 0.364 | | 1.29 | | | 0.198 | | | | 0.198 | | | |  | |  | |  | |  | | |  |  |
| AUDIT, the Alcohol Use Disorders Identification Test; OCDS, Obsessive Compulsive Drinking Scale; ADS, Alcohol Dependence Scale | | | | | | | | | | | | | | | | | | | | | | | | | |  | |  |
| ^a^Hap-Freq, estimated frequency of the haplotype in the pool of all participants. | | | | | | | | | | | | | | | | | | |  | |  | |  | |  |  | |  |
| ^b^Hap-Score, score for the haplotype. | | | | | |  | | |  | | | |  | | | | | |  | |  | |  | |  |  | |  |
| ^c^Asymptotic chi-square *p*-value. | | | | | |  | | |  | | | |  | | | | | |  | |  | |  | |  |  | |  |
| ^d^Simulated *p*-value. | | | |  | |  | | |  | | | |  | | | | | |  | |  | |  | |  |  | |  |

| **Table** **S5** The effects of haplotype on impulsivity behavioral task results. | | | | | | | | | | | | | |  | |  | |  | |  |  |  |
| --- | --- | --- | --- | --- | --- | --- | --- | --- | --- | --- | --- | --- | --- | --- | --- | --- | --- | --- | --- | --- | --- | --- |
| Task | Block | | | | Hap-Freq^a^ | | Hap-Score^b^ | | Crude *p*^c^ | Sim.*p*^d^ | | Global-stat | df | | *p* | | Global simulated *p* | |  |  |  |  |
| SSRT | 1 (rs6473797-rs702764) | | | |  | |  | |  |  | | 0.175 | 3 | | 0.982 | | 0.956 | |  |  |  |  |
|  | G | A |  | | 0.321 | | -0.259 | | 0.796 | 0.801 | |  |  | |  | |  | |  |  |  |  |
|  | G | G |  | | 0.0584 | | 0.107 | | 0.915 | 0.91 | |  |  | |  | |  | |  |  |  |  |
|  | A | A |  | | 0.619 | | 0.218 | | 0.828 | 0.829 | |  |  | |  | |  | |  |  |  |  |
|  | 2 (rs495491-rs1799971-rs609148) | | | |  | |  | |  |  | | 3.55 | 5 | | 0.616 | | 0.431 | |  |  |  |  |
|  | A | A | G | | 0.432 | | -0.923 | | 0.356 | 0.359 | |  |  | |  | |  | |  |  |  |  |
|  | G | A | G | | 0.129 | | -0.662 | | 0.508 | 0.509 | |  |  | |  | |  | |  |  |  |  |
|  | A | A | A | | 0.0451 | | -0.0223 | | 0.982 | 0.982 | |  |  | |  | |  | |  |  |  |  |
|  | G | A | A | | 0.015 | | 1.09 | | 0.274 | 0.231 | |  |  | |  | |  | |  |  |  |  |
|  | A | G | G | | 0.375 | | 1.20 | | 0.231 | 0.233 | |  |  | |  | |  | |  |  |  |  |
| DDT (k value) | 1 (rs6473797-rs702764) | | | |  | |  | |  |  | | 2.07 | 3 | | 0.558 | | 0.533 | |  |  |  |  |
|  | G | A |  | | 0.322 | | -1.10 | | 0.269 | 0.27 | |  |  | |  | |  | |  |  |  |  |
|  | A | A |  | | 0.62 | | 0.553 | | 0.58 | 0.581 | |  |  | |  | |  | |  |  |  |  |
|  | G | G |  | | 0.0553 | | 0.87 | | 0.385 | 0.383 | |  |  | |  | |  | |  |  |  |  |
|  | 2 (rs495491-rs1799971-rs609148) | | | |  | |  | |  |  | | 3.66 | 5 | | 0.599 | | 0.576 | |  |  |  |  |
|  | A | G | G | | 0.375 | | -0.566 | | 0.571 | 0.573 | |  |  | |  | |  | |  |  |  |  |
|  | A | A | G | | 0.433 | | -0.435 | | 0.664 | 0.665 | |  |  | |  | |  | |  |  |  |  |
|  | G | A | G | | 0.129 | | 0.589 | | 0.556 | 0.556 | |  |  | |  | |  | |  |  |  |  |
|  | G | A | A | | 0.0154 | | 1.19 | | 0.235 | 0.24 | |  |  | |  | |  | |  |  |  |  |
|  | A | A | A | | 0.0434 | | 1.24 | | 0.215 | 0.215 | |  |  | |  | |  | |  |  |  |  |
| BART | 1 (rs6473797-rs702764) | | | |  | |  | |  |  | | 2.74 | 3 | | 0.434 | | 0.431 | |  |  |  |  |
|  | G | G |  | | 0.0572 | | -0.822 | | 0.411 | 0.412 | |  |  | |  | |  | |  |  |  |  |
|  | A | A |  | | 0.62 | | -0.811 | | 0.418 | 0.418 | |  |  | |  | |  | |  |  |  |  |
|  | G | A |  | | 0.32 | | 1.39 | | 0.165 | 0.164 | |  |  | |  | |  | |  |  |  |  |
|  | 2 (rs495491-rs1799971-rs609148) | | | |  | |  | |  |  | | 3.48 | 5 | | 0.627 | | 0.616 | |  |  |  |  |
|  | G | A | A | | 0.015 | | -0.946 | | 0.344 | 0.346 | |  |  | |  | |  | |  |  |  |  |
|  | G | A | G | | 0.13 | | -0.946 | | 0.344 | 0.344 | |  |  | |  | |  | |  |  |  |  |
|  | A | G | G | | 0.371 | | 0.216 | | 0.829 | 0.829 | |  |  | |  | |  | |  |  |  |  |
|  | A | A | G | | 0.439 | | 0.258 | | 0.796 | 0.797 | |  |  | |  | |  | |  |  |  |  |
|  | A | A | A | | 0.041 | | 0.619 | | 0.536 | 0.539 | |  |  | |  | |  | |  |  |  |  |
| SSRT, stop signal reaction time; DDT, delayed discount test; BART, balloon analog task | | | | | | | | | | | | | | | |  | |  | |  |  |  |
| ^a^Hap-Freq, estimated frequency of the haplotype in the pool of all participants. | | | | | | | | | | | | | |  | |  | |  | |  |  |  |
| ^b^Hap-Score, score for the haplotype. | | | | | |  | |  | | |  | | |  | |  | |  | |  |  |  |
| ^c^Asymptotic chi-square *p*-value. | | | | | |  | |  | | |  | | |  | |  | |  | |  |  |  |
| ^d^Simulated *p*-value. | | | |  | |  | |  | | |  | | |  | |  | |  | |  |  |  |
